# Cholesterol Induces Specific Spatial and Orientational Order in Cholesterol/Phospholipid Membranes

**DOI:** 10.1371/journal.pone.0011162

**Published:** 2010-06-17

**Authors:** Hector Martinez-Seara, Tomasz Róg, Mikko Karttunen, Ilpo Vattulainen, Ramon Reigada

**Affiliations:** 1 Department of Physical Chemistry and Institut de Química Teòrica i Computacional (IQTCUB), Universitat de Barcelona, Barcelona, Spain; 2 Department of Physics, Tampere University of Technology, Tampere, Finland; 3 Department of Applied Mathematics, The University of Western Ontario, London, Ontario, Canada; 4 MEMPHYS-Center for Biomembrane Physics, University of Southern Denmark, Odense, Denmark; 5 Department of Applied Physics, Aalto University School of Science and Technology, Espoo, Finland; Johns Hopkins School of Medicine, United States of America

## Abstract

**Background:**

In lipid bilayers, cholesterol facilitates the formation of the liquid-ordered phase and enables the formation of laterally ordered structures such as lipid rafts. While these domains have an important role in a variety of cellular processes, the precise atomic-level mechanisms responsible for cholesterol's specific ordering and packing capability have remained unresolved.

**Methodology/Principal Findings:**

Our atomic-scale molecular dynamics simulations reveal that this ordering and the associated packing effects in membranes largely result from cholesterol's molecular structure, which differentiates cholesterol from other sterols. We find that cholesterol molecules prefer to be located in the second coordination shell, avoiding direct cholesterol-cholesterol contacts, and form a three-fold symmetric arrangement with proximal cholesterol molecules. At larger distances, the lateral three-fold organization is broken by thermal fluctuations. For other sterols having less structural asymmetry, the three-fold arrangement is considerably lost.

**Conclusions/Significance:**

We conclude that cholesterol molecules act collectively in lipid membranes. This is the main reason why the liquid-ordered phase only emerges for Chol concentrations well above 10 mol% where the collective self-organization of Chol molecules emerges spontaneously. The collective ordering process requires specific molecular-scale features that explain why different sterols have very different membrane ordering properties: the three-fold symmetry in the Chol-Chol organization arises from the cholesterol off-plane methyl groups allowing the identification of raft-promoting sterols from those that do not promote rafts.

## Introduction

Cholesterol (Chol) is the most common lipid component in animal cell membranes [1]. It largely determines permeability, fluidity, and mechanical properties of the membranes [2]. It increases the order of fluid-phase phospholipid acyl chains, giving rise to the formation of the liquid-ordered (lo) phase [3], [4]. Through this process, it is also involved in the formation of highly ordered nano-scale membrane domains called lipid rafts [5], [6] which play an important role in numerous cellular functions [7]. Here we focus on a related issue, the nano-scale organization of cholesterol-containing membranes.

Experimental and theoretical studies have shown that Chol has the tendency to form regularly distributed lateral structures [8], [9]. On the macroscopic level they are known to correspond to the lo phase, but the actual atomic-level lipid organization and the associated physical mechanisms responsible for this organization have remained unclear. Conceptual models like the Condensed Complex model [10] (based on low-energy lipid-Chol complexes), the Superlattice model [11] (based on the existence of extended ordered distributions) and the Umbrella model [12] (based on the fact that cholesterol has a small hydrophilic headgroup which cannot shield its hydrophobic ring system from water) have been proposed for describing the formation of these structures. They are, however, not able to differentiate cholesterol from other sterols. There is an increasing amount of evidence that the organizing mechanisms are specific to the structure of cholesterol. This view is supported, e.g., by the fact that cholesterol and the structurally very similar ergosterol [13] are known to enable formation of rafts, while many other sterols lack this capability [14]–[18].

**Figure 1:**
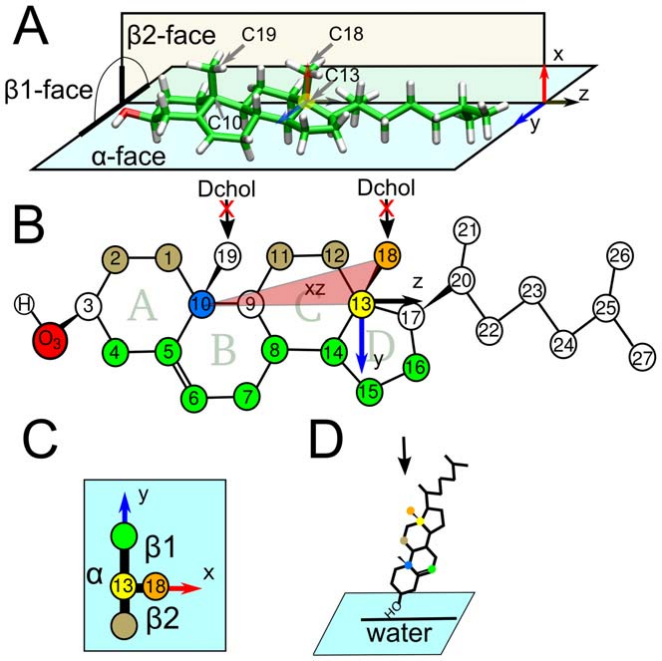
Three-dimensional structure of cholesterol: the α- and ß-faces. Panel (A) shows the three-dimensional structure of cholesterol. The positions of cholesterol's flat α-face and the rough β1- and β2-subfaces due to the two out-of-plane methyl groups C18 and C19 are shown. Panel (B) provides the numbering of the carbon atoms. Panels (A) and (B) show the reference axes used in this article. The origin of the axis is C13 colored in yellow. The vector between C13 (yellow) and C18 (orange) points along the *x*-axis. The triangle between C18 (orange), C13 (yellow) and C10 (blue) is in the *xz*-plane and is depicted in red. This plane divides the molecule between the β1-face *y*>0 and β2-face *y*<0. The atoms of cholesterol that belong to the β1-face are shown in brown, while those in the β2-face are colored in green. Panel (C) shows a schematic view of cholesterol as a projection in the reference *xy*-plane from the terminal acyl chain towards the head as shown in Panel (D) by the arrow. The other sterol analyzed in this work, Dchol, is obtained by the removal of the off-plane methyl groups C18 and C19 as shown in Panel (B).

Cholesterol is composed [1] of a semi-rigid tetracyclic ring system with a hydroxyl group and a short 8-carbon atom chain attached to carbon C17 as shown in Figures 1A and 1B. The ring system forms an asymmetric planar structure with two off-plane methyl substituents (C18, C19) defining the so-called rough side often called as the β-face (Figures 1A-D). The smooth α-face has no methyl substituents. The role and the importance of this asymmetry have been studied extensively using atomistic molecular dynamics simulations. For reviews, see Refs. [19] and [20].

Our early studies have shown that cholesterol's ability to induce order is strongly related to the non-favorable interaction between the off-plane cholesterol methyl groups in the β-face and double bonds in lipid acyl chains [21], [22]. As a consequence, order is enhanced in saturated bilayers. The off-plane methyl groups are also known to modulate cholesterol's tilt angle in a bilayer. The tilt angle has been shown to be a fundamental characteristic in describing different sterols' ordering capabilities [17], [23].

Other detailed studies have also shown that off-plane methyl groups decrease chain order and packing of saturated lipids facing a cholesterol's rough β-face [24], [25]. In contrast, strong ordering has been observed in saturated chains located next to the cholesterol's smooth α-face. In the case of unsaturated lipids the effects of the two cholesterol faces are reversed: chains located next to the rough β-face are more ordered than those located next to the smooth α-face [26]. This observation is in agreement with the simulation results of Pandit and Scott [27]. They showed that the cholesterol α-face interacts preferentially with saturated sphingomyelin molecules and that the β-face tends to interact with unsaturated DOPC lipids, with a preference of cholesterol for being located at the border of liquid-ordered (raft-like) domains [27].

Despite numerous experimental and simulation studies the detailed physical origin of how cholesterol is unique in promoting the formation of the liquid-ordered phase and what is the in-plane membrane structure emerging from the interactions of cholesterol with other lipids in the Chol-rich domain have remained unresolved [19], [20], [28].

In this article we show how the specific molecular structure of cholesterol determines cholesterol-induced lateral lipid organization. To do this we performed atomistic molecular dynamics simulations of different membranes composed of cholesterol and both saturated and unsaturated phospholipids. Additional simulations with a cholesterol-like sterol lacking the methyl groups in the β-face (Dchol, see Figure 1B) were also carried out to specify the effects of the off-plane methyl groups. As our main results, we observed the preference of cholesterol molecules to be located in the second coordination shell (∼1 nm), thus avoiding direct cholesterol-cholesterol contact, and that the cholesterol off-plane methyl groups induce a three-fold symmetrical arrangement of proximal cholesterol molecules. The distribution of relative orientations between two cholesterol molecules separated by a single lipid molecule displays a clear non-random pattern revealed by the existence of preferred relative Chol-Chol orientations. In all these findings, the role of the cholesterol off-plane methyl groups is observed to be fundamental. The biological significance of our findings is discussed at the end of this article.

## Methods

We performed atomistic molecular dynamics (MD) simulations of membrane systems composed of fully saturated distearoyl phosphatidylcholine (DSPC) and di-unsaturated dioleoyl phosphatidylcholine (DOPC) in the fluid phase with 10, 20, 30, 40 and 50 mol% of Chol. DSPC has two fully saturated 18∶0 stearoyl chains, whereas in DOPC the acyl chains have the same length but both the *sn*-1 and *sn*-2 chains are *cis* monounsaturated. For comparison, additional simulations were performed with 20 and 40 mol% of ‘flat cholesterol’ (Dchol) [23], [29], whose structure corresponds to that of Chol where the C18 and C19 methyl groups have been removed. Figure 1B shows the structural differences between Chol and Dchol. Each membrane contained 128 PCs and the corresponding number of sterol molecules (14, 32, 56, 86 and 122).

First, we constructed the DOPC system containing 50 mol% of cholesterol starting from a membrane containing four lipids and four cholesterols in each leaflet. To minimize artificial ordering, cholesterol molecules were randomly rotated in the DOPC matrix. This patch was replicated 4×4 in the membrane (*xy*) plane. Next, 6186 water molecules were added into the system. The final configuration of a 10 ns simulation was used as a starting configuration for the DSPC system with 50 mol% of cholesterol. The same configuration was used for DOPC with 40 mol% of cholesterol after randomly subtracting the appropriate number of cholesterol molecules. This procedure was repeated to construct the other bilayers. The simulation times of each system totaled 200–300 ns including 20 ns of equilibration. The total simulated time scale exceeded 3 µs. To ensure that 20 ns was enough for equilibration, we computed additional structural parameters (such as radial distribution functions) and autocorrelation functions averaged over different time windows. The results showed that the computed quantities had no drift after 20 ns (data not shown).

The simulations were performed using the GROMACS software package [30], [31]. We used the standard united-atom force-field parameters that have been extensively tested and verified for saturated dipalmitoylphosphatidylcholine (DPPC) molecules (see e.g., Refs. [32] and [33], and references therein). The partial charges were taken from the underlying model description [34]. We used the description by Bachar et al. [35], [36] for the double bonds in the DOPC acyl chains. This is important since the standard united-atom GROMOS87 force field [37] uses only a simple improper torsion with corrections for the adjacent dihedrals to parameterize the *cis* double bond. The description of Bachar et al. takes into account the *skew* states in the vicinity of a double bond. It has been shown in independent studies that the double bond description of Bachar et al. provides an important correction in both pure [35], [36], [38], [39] and cholesterol containing [36] bilayers. The Simple Point Charge (SPC) model [40] was used for water. For cholesterol, we used the description of Holtje et al [41] as described in detail in Ref. [23]. The SETTLE algorithm [42] was used to preserve the bond lengths in water molecules, while the lipid bond lengths were constrained using the LINCS algorithm [43]. A single 1.0 nm cutoff distance was used for Lennard-Jones interactions [33]. Long-range electrostatic interactions were computed using the particle-mesh Ewald method [44], [45] with a real space cut-off of 1.0 nm, spline interpolation of order 6 and direct sum tolerance of 10^−5^. Periodic boundary conditions with the usual minimum image convention were used in all three directions and the time step was set to 2 fs.

The simulations were carried out in the NpT (constant particle number, pressure and temperature) ensemble at p = 1 atm and T = 338 K. The selected temperature is above the main phase transition temperature of DSPC (T_m_ = 328 K) – the highest among the studied lipid species [46]. Temperature and pressure were controlled by using the weak coupling method [47] with the relaxation times set to 0.6 and 1.0 ps, respectively. The temperatures of the solute and solvent were controlled independently and the pressure coupling was applied separately in the bilayer plane (*xy)* and in the perpendicular direction (*z*).

The simulation protocol used in this work has been successfully applied in numerous previous MD simulations [6], [17], [21], [35], [48]. In our recent studies [21], [36] the obtained values for global structural membrane properties such as the average area per lipid and membrane thickness have been well in line with experimental data [49]–[51] for pure DOPC and DSPC bilayers, and for related bilayers with 20 mol% of Chol. The dependence of global membrane properties on cholesterol concentration (10–50 mol% of Chol) and the related recent experimental data [52]–[54] will be discussed elsewhere. Here the focus is on the characterization of the local molecular organization of cholesterol-phospholipid structures.

## Results

### Cholesterol molecules avoid being located in adjacent positions

**Figure 2:**
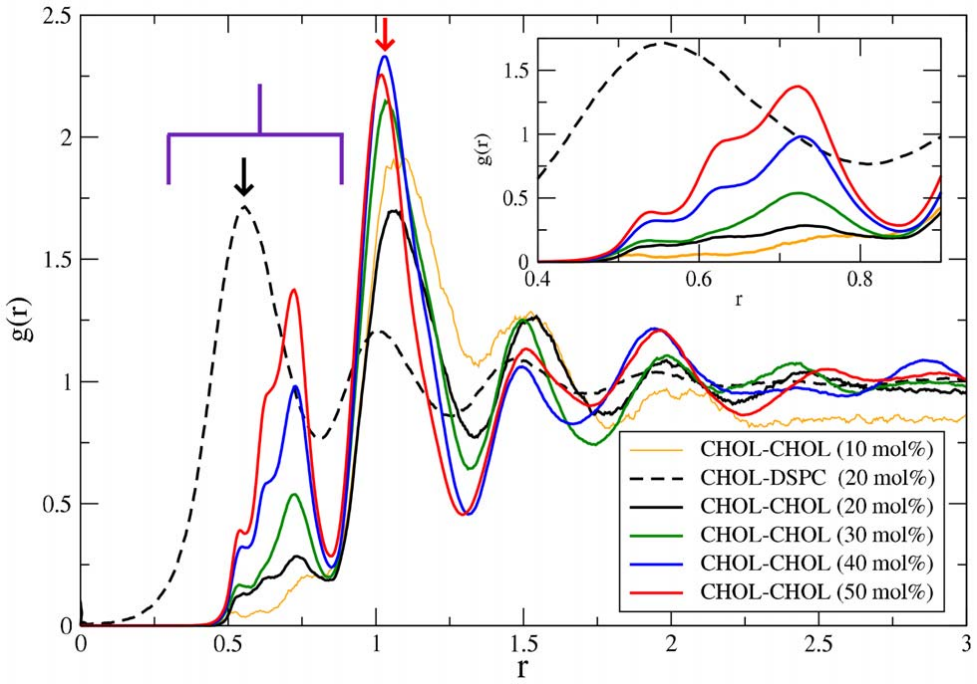
Radial distribution of lipids and cholesterol around cholesterol molecules. Pair correlation functions, g(r), for Chol-Chol (solid) and Chol-DSPC (dashed) pairs in the simulated DSPC/Chol membranes at different Chol concentrations. The color code indicates the cholesterol content: 10 mol% (yellow), 20 mol% (black), 30 mol% (green), 40 mol% (blue) and 50 mol% (red). Data for Chol-DSPC pairs is only provided for the system with 20 mol% of Chol. The radial distance from the center of mass of a Chol molecule is denoted by r. The inset shows a magnification of the first coordination shell. For Chol-Chol, the peak increases with increasing Chol concentration. The shapes of Chol-Chol peaks at high concentrations clearly show that they are composed of 3 overlapping peaks with maxima at approximately 0.45, 0.65 and 0.73 nm.

We start by analyzing the pair correlation functions, *g*(*r*), for DPSC/Chol membranes at different Chol concentrations. The variable *r* stands for the radial distance from the center of mass of a cholesterol molecule and *g*(*r*) measures density variation with respect to the average density as a function of distance. The results are shown in Figure 2. For a Chol-DSPC pair (data is provided only for 20 mol% Chol), *g*(*r*) has its main peak at a distance of ∼0.5 nm. As this is also the first peak, it corresponds to the first coordination shell. In contrast, the main peak for Chol-Chol pair correlations is located at ∼1.0 nm. This is the second peak and hence corresponds to the second coordination shell. The physical interpretation of this observation is that cholesterols avoid being located in adjacent positions [6], [55]. This behavior is common in all systems with moderate cholesterol concentrations. Only at high cholesterol concentrations (>30 mol%) does the occurrence of close contacts (<0.8 nm) become relevant (see Table 1) since then the membrane is crowded with cholesterol molecules.

**Table 1:** Ratio of cholesterol density averaged inside the first (0.4–0.8 nm) and the second (0.8–1.1 nm) coordination shells with respect to the total mean density.

DSPC +	10% Chol	20% Chol	30% Chol	40% Chol	40% Dchol	50% Chol
0.4–0.8 nm	0.253	0.228	0.277	0.480	0.441	0.670
0.8–1.1 nm	1.49	1.24	1.40	1.46	1.52	1.44

DOPC +	10% Chol	20% Chol	30% Chol	40% Chol	40% Dchol	50% Chol
0.4–0.8 nm	0.300	0.368	0.364	0.516	0.574	0.700
0.8–1.1 nm	1.51	1.39	1.46	1.47	1.55	1.41

We have used 1.1 nm to define the range of the second coordination shell around cholesterol. To avoid any contribution from the third coordination shell, all measures of the second coordination shell were conducted between 0.8–1.1 nm.

The inset in Figure 2 shows that the peaks observed in the first Chol-Chol coordination shell (0.4–0.8 nm) at high Chol concentrations are due to a superposition of three closely spaced individual peaks. This is a clear signature of the existence of three distinct preferential Chol-Chol pairs within the first coordination shell, and warrants a more detailed analysis using angular information in addition to the radial distance. This analysis will be provided in the next section.

Figure 2 also shows that the second coordination shell does not have any indications of possible superposition of multiple maxima even at high Chol concentrations. However, in the angular analysis presented below preferential directions are also clearly observed, so it seems that in this case the related effects have been averaged in the angular summation.

In addition, Table 1 shows that the number of Chol-Chol contacts increases systematically in the first shell upon increasing Chol concentration. Such behavior is not observed for the second coordination shell in which the averaged cholesterol density remains rather constant. Similar behavior is found for DOPC but with slightly higher occurrence of direct Chol-Chol contacts (see Table 1). This is expected since cholesterol has a higher affinity for saturated than for unsaturated lipids.

The absence of direct Chol-Chol contacts at low Chol concentrations may be due to the small hydrophilic headgroup of cholesterol; a close contact of two sterol molecules is not energetically favorable since such a contact would prefer high curvature in order to shield the hydrophobic cholesterol ring system. In a planar bilayer, this would lead to the exposition of the hydrophobic membrane core to water and thus be energetically very costly. The entropic cost of having two rigid cholesterol molecules in direct contact is also high. It is thus favorable to have a PC molecule with a flexible chain next to a cholesterol molecule. Such an arrangement is one of the key ingredients of the Umbrella model of Huang and Feigenson [12]. In agreement with the above, in the Umbrella model the free-energy cost of covering a Chol-Chol dimer is much higher than that of covering a single cholesterol molecule. Similar to the observations in Chol containing bilayers, direct Dchol-Dchol contacts are also very unfavorable (see Table 1).

Finally, due to crowding at large sterol fractions, direct Chol-Chol contacts are unavoidable and can be responsible for the jumps in the cholesterol chemical potential at certain concentrations in PC/Chol bilayers [8]. The above differences in free-energy may explain the jumps in the cholesterol chemical potential observed experimentally in PC membranes containing large amount of cholesterol (>30 mol%) [8].

### Cholesterol molecules form a three-fold symmetric arrangement

Next we analyze lateral ordering of lipid molecules by computing the in-plane two-dimensional (2D) average density. We computed the total densities instead of probability densities as in the case of *g*(*r*) above. Since we are computing 2D density distributions, we obtain angular information in contrast to the angularly averaged one-dimensional *g*(*r*).

**Figure 3:**
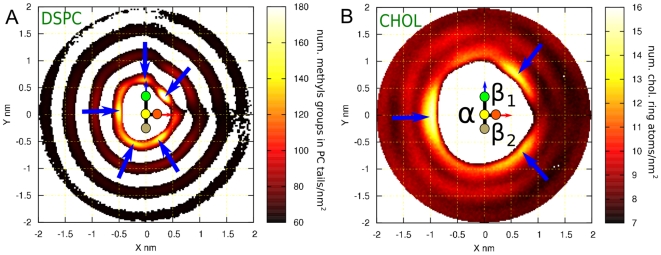
In-plane distribution of lipids and cholesterols around cholesterols in DSPC bilayer with 20 mol% Chol. Two-dimensional density distributions for (A) DSPC acyl chains and (B) Chol molecules around a tagged Chol. Data shown is for DSPC bilayer with 20 mol% Chol. Each plot shows a schematic representation of the tagged cholesterol as described in Figure 1. The different faces of cholesterol can be distinguished in both panels: the smooth α-face corresponds to the region *x*<0 and the rough β-face to *x*>0. The β-face is divided into two sub-faces: β1 for *y*>0 and β2 for *y*<0. Panel (A) shows the density distribution of methyl groups of both acyl chains per nm^2^. The first coordination shell is located around 0.5 nm from the center of the tagged molecule. The five preferred locations for PC chains are marked by the blue arrows. Cholesterol's ability to induce order is demonstrated by three additional less structured coordination shells. Panel (B) shows the density distribution of atoms in the ring of the cholesterol molecule (17 atoms per cholesterol molecule). Panel (B) also shows that cholesterols avoid the first coordination shell covered by the PC acyl chains. Instead a clear and structured second coordination shell appears. The three emerging peaks are marked with blue arrows. Each peak is on a different face (α, β1 and β2) displaying a three-fold symmetry.

The analysis was performed the following way: for each cholesterol molecule the origin was centered at the C13 group (Figure 1A). Then, the *x-*axis was defined by the C13–C18 direction and the *xz-*plane was chosen to include the C13–C10 vector (Figure 1B). Using this frame of reference for each cholesterol molecule, we computed the averaged 2D-density distributions for methyl groups in PC acyl chains (Figure 3A) and for the atom groups in the cholesterol ring (Figure 3B) projected in the reference *xy*-plane in both cases.

Figure 3 shows the distributions for a DSPC membrane with 20 mol% Chol. The first shell located at ∼0.5 nm (see also Figure 2) is occupied almost exclusively by PC molecules, while the second one at ∼1 nm (see also Figure 2) is where Chol molecules reside. In both cases ordering is strongly suppressed in the positive *x*-direction. That is due to the steric effects of the C18 and C19 off-plane groups on the rough β-face of cholesterol.

In all cases we observed at least three coordination shells for PC acyl chains. For the saturated DSPC moiety the number of shells is increased to four or even five (data not shown). For example, Figure 3A shows four clear coordination shells in the DSPC 20 mol% cholesterol system as a consequence of Chol's strong ordering ability and its affinity for saturated moieties [21].

The distance between each coordination shell is about ∼0.5 nm. A closer inspection of the first coordination shell reveals that PC acyl chain ordering is anisotropic in the bilayer plane and five clearly defined maxima appear (Figure 3A). Although this is true for all the analyzed systems, some of these maxima merge in the DOPC systems leaving only three preferred locations (not shown). This behavior can be understood in terms of the more fluid nature shown by membranes constituted by unsaturated lipids, possibly denoting a less restricted environment. Similar analysis of DOPC-Chol and SM-Chol interactions by Pandit et al. in a ternary mixture did not show the same ordering symmetry found here [27]. There are two reasons for such a discrepancy. One is the presence of two different components in the cholesterol surroundings. Another reason is the complexity of a ternary mixture. Ternary mixtures require long relaxation times than two-component systems and it has been seen in other lipid systems that the behavior of a ternary mixture cannot be derived from that of a binary one [56].

The arrangements of cholesterol molecules with respect to each other are even more interesting. Clear anisotropy and well-defined triangular symmetry with a clear peak at the α-face and two maxima at the β-face are observed. The β-face maxima split further into the two sub-faces β1 and β2 (see Figures 1C and 3B). The 2D-density plots for DOPC bilayers display similar trends as for DSPC, but with slightly less well-defined peaks and coordination shells (not shown). That concurs with the established fact that Chol orders saturated chains more than unsaturated ones [21].

The presence of this three-fold symmetry was previously shown by a visualization of isosurfaces of lipids and cholesterols around a cholesterol molecule by Pitman et al [57]. They found, however, that cholesterol was often located in the first coordination shell. This is probably due to the use of polyunsaturated lipids. They have less ability to pack laterally with cholesterol molecules as compared to monounsaturated lipids used in this work. Our simulations at high cholesterol concentrations also display density peaks in the first coordination shell although at different positions in the 2D-averaged density profiles (at angle ranges [−10°–10°], [80°–100°] and [240°–270°]; see Supporting Information Text S1 and Figures S1 and S2). These three preferred locations correspond to the three convoluted peaks observed in the first coordination shell in Figure 2.

The importance of off-plane cholesterol methyl groups to lateral ordering anisotropy was confirmed by computing the 2D-density functions for membranes containing Dchol instead of Chol. The purpose of simulating this ‘flat’ sterol stems from the biosynthetic pathway of cholesterol [58]. The first sterol on this pathway is lanosterol, which has a structure similar to that of cholesterol except for three additional methyl groups located at both faces of lanosterol. The additional methyl groups are gradually removed along the biosynthetic process. Since the cholesterol biosynthetic pathway is thought to reflect evolutionary changes that finally result in the optimized cholesterol structure, one may think that further optimization could be achieved by removing the two remaining methyl groups from cholesterol. It leads to the (non-natural) Dchol sterol molecule used in some of our simulations to determine the role of the off-plane methyl groups. Here, the abbreviation Dchol indeed stands for the flat cholesterol instead of dihydrocholesterol which often is given the same abbreviation.

**Figure 4:**
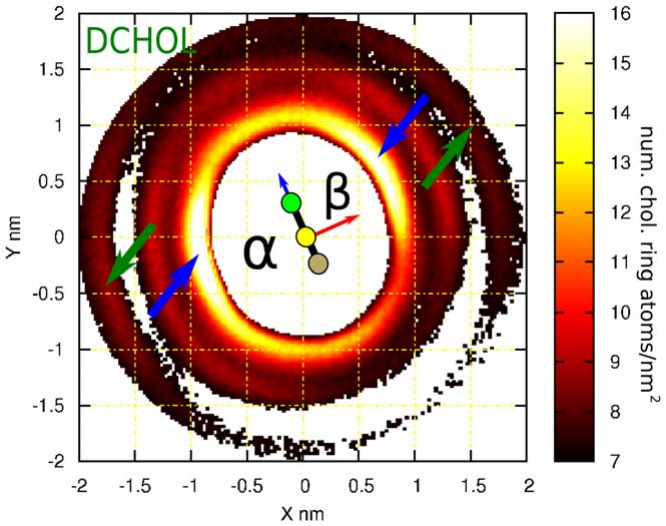
In-plane distribution of lipids and Dchol around Dchol in DSPC bilayer with 20 mol% Dchol. The two-dimensional average density distribution for Dchol molecules around a tagged Dchol. This corresponds to the density distribution of atoms in the ring of the Dchol molecules (15 atoms per Dchol molecule). The data presented corresponds to a DSPC bilayer with 20 mol% Dchol. A schematic representation of the tagged Dchol is shown at the center. As Dchol does not possess out-of-plane methyl groups, it is represented by a simple rod. As the presence of C18 is required to define the axes (see Figure 1), the missing methyl groups in Dchol molecule were reconstructed assuming tetrahedral geometry and thus α- and β- faces can be recognized. A comparison between the two sides of Dchol shows that their behavior is similar to behavior of the smooth Chol face. Similar to the behavior of Chol, no Dchol is seen in the first coordination sphere. On each side of the second coordination shell a peak facing the rod is observed (marked with blue arrows). Some structure is still present in the outer coordination shell (∼1.8 nm) displaying peaks collinear with the previous ones (green arrows). This reflects a strong preference to form linear Dchol-Dchol structures.

**Figure 5:**
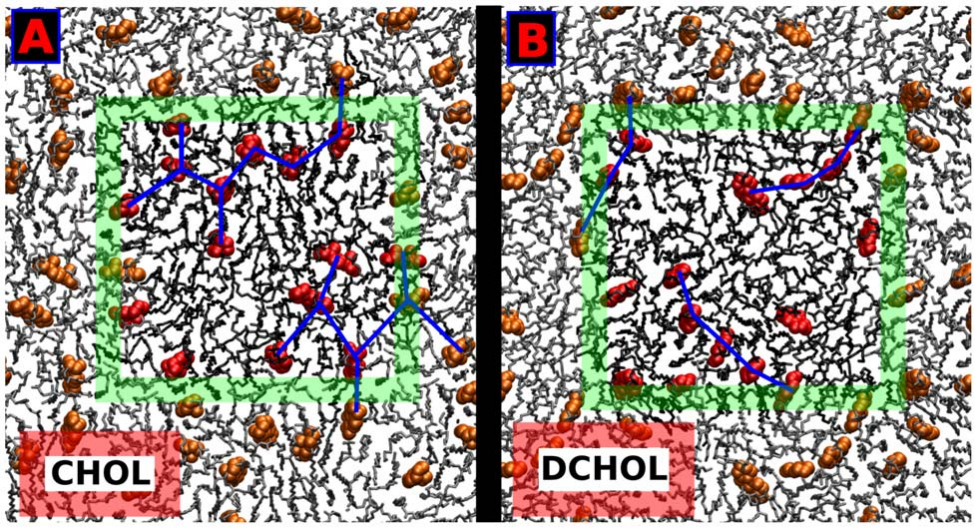
Configurations of Chol and Dchol molecules in the membrane. Top view of an equilibrated configuration of (A) the DSPC/Chol bilayer and (B) the DSPC/Dchol system at 20 mol% of sterol in both cases. Only one leaflet is presented for clarity. PC molecules are shown as black sticks and sterols using space-filling representation in red color. The simulation box is shown by the green square with periodic images plotted around it. Sterols in periodic images are show in orange (instead of red as in the simulation box) and lipids in gray. Panel (A) shows triangular connections between neighboring Chol molecules. The blue lines between Chols form triangular patterns. Panel (B) displays the linear connections between Dchol molecules. This fundamental difference is due to the missing out-of-plane methyl groups in the Dchol molecule.

To define the axes in an equivalent manner as for Chol the positions of missing methyl groups in Dchol molecule were reconstructed assuming tetrahedral geometry. As a consequence α- and β- faces can be recognized although both faces are smooth for Dchol. For both PC acyl chain and sterol distributions, a two-fold symmetry is observed with one broad peak at each side of the sterol plane, Figure 4. Cholesterol distribution shows a clear preference to form triangular arrangements (see Figure 5A) as can be seen from the 2D-density probability functions in Figure 3B. Dchol, instead, displays linear-like patterns (Figure 5B) as a result of the two-fold symmetry displayed in the 2D-density distribution functions in Figure 4. In Figure 5, we present snapshots of one leaflet of the DSPC/Chol and DSPC/Dchol membranes. Triangular ordering of Chol and a linear array of Dchol are clearly identified. Although lanosterol is not considered in our simulations, we predict that it would not show three-fold symmetry due to the off-plane methyl groups placed at the α-face. Since the C18 cholesterol methyl group has been shown to disturb membrane structure more than C19 which is closest to the headgroup [21], we expect that the methyl group attached to C14 in lanosterol would disturb the membrane more than the methyl groups attached to the C4 position closer to the headgroup. Hence, we predict some sort of four-fold symmetry for lanosterol-containing membranes.

### Lipid molecules display orientational order

In this section we show that phospholipids prefer specific positions in the first Chol coordination shell. This is an important finding as it also influences ordering in the second shell as will be discussed in detail below.

**Figure 6:**
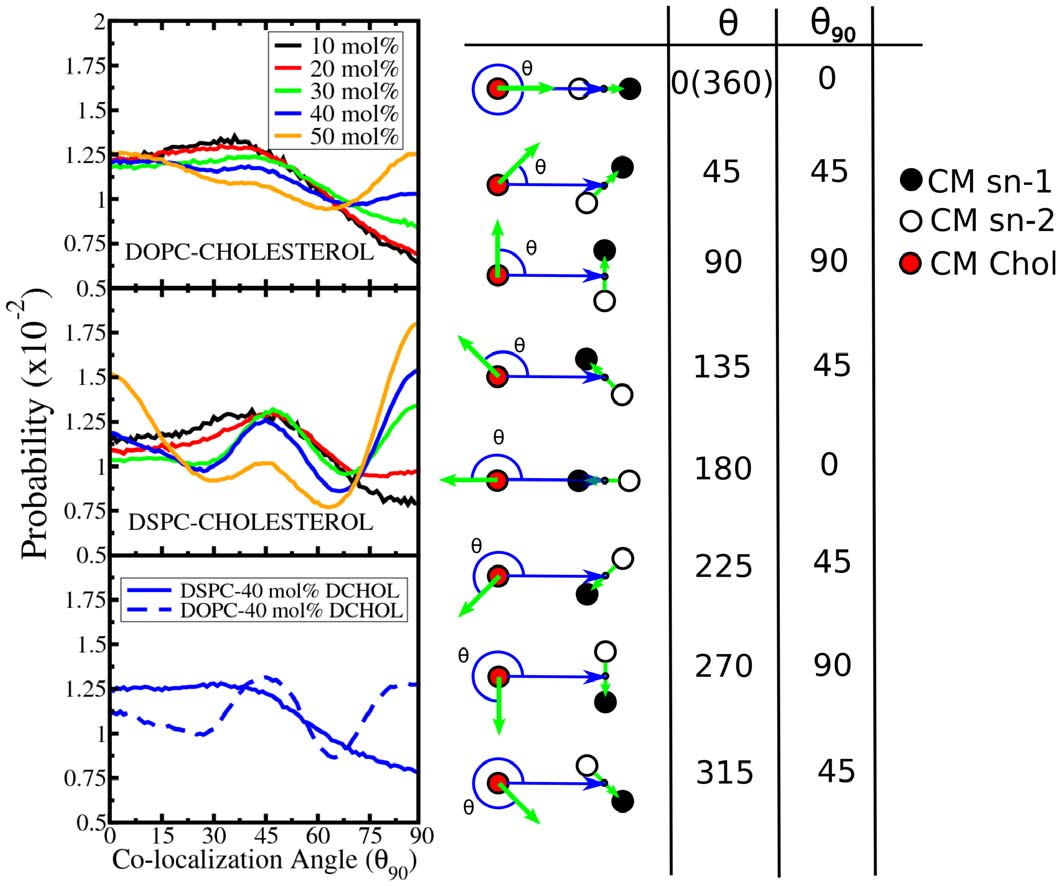
Lipid orientation around Chol and Dchol molecules in the second coordination shell. Distribution profiles of the co-localization angles for all simulated systems for lipids inside the first coordination shell of Chol. Color code indicates sterol content. The graphical representation of the co-localization angle is provided next to the graphs. Green arrows denote the vector joining the centers of mass of the two acyl chains and the blues ones the vector between the center of mass of the cholesterol molecule and the mid-point of the previous vector. The red, white and black dots are the centers of masses of the *sn-1* acyl chain, the *sn-2* acyl chain and the Chol ring, respectively. The θ_90_ representation is used to represent the co-localization angle in all graphs (θ_90_ = 0° corresponds to collinear, θ_90_ = 45° to diagonal and θ_90_ = 90° to ‘facing’ orientations).

Initially, we calculated the center of mass of each acyl chain for a given PC molecule with at least one of them in the first coordination shell. They were found to have an average separation between their *sn*-1 and *sn*-2 acyl chains of (0.55−0.85±0.2) nm depending on the considered moiety and membrane system. Next, we analyzed the orientation of PC molecules in the first coordination shell with respect to their neighboring cholesterol molecules. Our analysis was performed as follows: 1) we selected PC molecules with the center of mass of at least one of their acyl chains in the first coordination shell (up to 0.75 nm from the center of mass) of a given Chol. 2) For these lipids we computed (a) the vector joining the centers of masses of the two acyl chains and (b) the vector between the center of mass of the cholesterol molecule and the mid-point of the above-calculated vector. This angle is denoted by θ. To better capture the symmetry properties, we used a 90 degree representation where both acyl chains are considered equivalent. Using this symmetry, the independent angles, labeled θ_90_, run up to 90 degrees and the remaining θ values are covered by symmetry. We call θ_90_ the “co-localization angle”. Its relation to θ is shown on the right hand side of Figure 6. θ_90_ = 0° corresponds to the situation in which all of the centers of masses are lined up (PC collinear to Chol). The angle θ_90_ = 90° corresponds to the situation in which the centers of masses corresponding to the lipid acyl chains are at equal distances from the Chol center of mass (‘facing’ orientation). Distributions of the “co-localization angle” are shown in Figure 6.

Figure 6 shows that the orientation of lipids around Chol molecules depends strongly on Chol concentration and on the type of PC lipids. DOPC promotes profiles with less structure (less selectivity) than DSPC. This is particularly clear at high cholesterol concentrations. The profiles show gradual changes upon varying cholesterol concentrations. At the concentration of 10 mol% of Chol, the profiles for both DOPC and DSPC are qualitatively similar favoring angles between 0–45 degrees. At larger Chol fractions, and particularly for DSPC membranes, the orientation profile displays preferential peaks at 0, 45 and 90 degrees. This is clearly observed for DSPC membranes (>20 mol% Chol) and to a lesser extent in DOPC systems (>30 mol% Chol). It seems plausible that there is a class of PC molecules that can be categorized by the preference for a 90° co-localization angle, especially at high cholesterol concentrations. Such lipids are likely sandwiched between two cholesterol molecules. Finally, the results for Dchol reveal that despite the small differences between the Chol and Dchol profiles (compare PC/Chol and PC/Dchol at 40 mol%), the off-plane methyls of Chol have little influence on lipid orientations that are determined mainly by the lipid moieties themselves.

A more detailed analysis shows that the orientations of PC molecules depend also on the side of the cholesterol molecule that is neighboring the analyzed chain. This is observed only for high cholesterol concentrations (>30 mol%) in DSPC bilayer and for 50 mol% in DOPC bilayer (see Supplementary Information Text S1 and Figure S3).

### Cholesterol molecules display orientational ordering

In our last analysis we focus on the orientational order of Chol. We have already seen that neighboring sterol molecules (corresponding to the 1 nm coordination shell) interact in such a way that a particular triangular symmetry appears in their relative spatial distribution. This occurs despite the fact that at least one PC molecule is intercalated between every two proximal Chol molecules. The planar structure of Chol and the off-plane methyl groups are fundamental for this organization. Moreover, our analysis shows that the relative orientation between two neighboring Chols display preferential angles.

**Figure 7:**
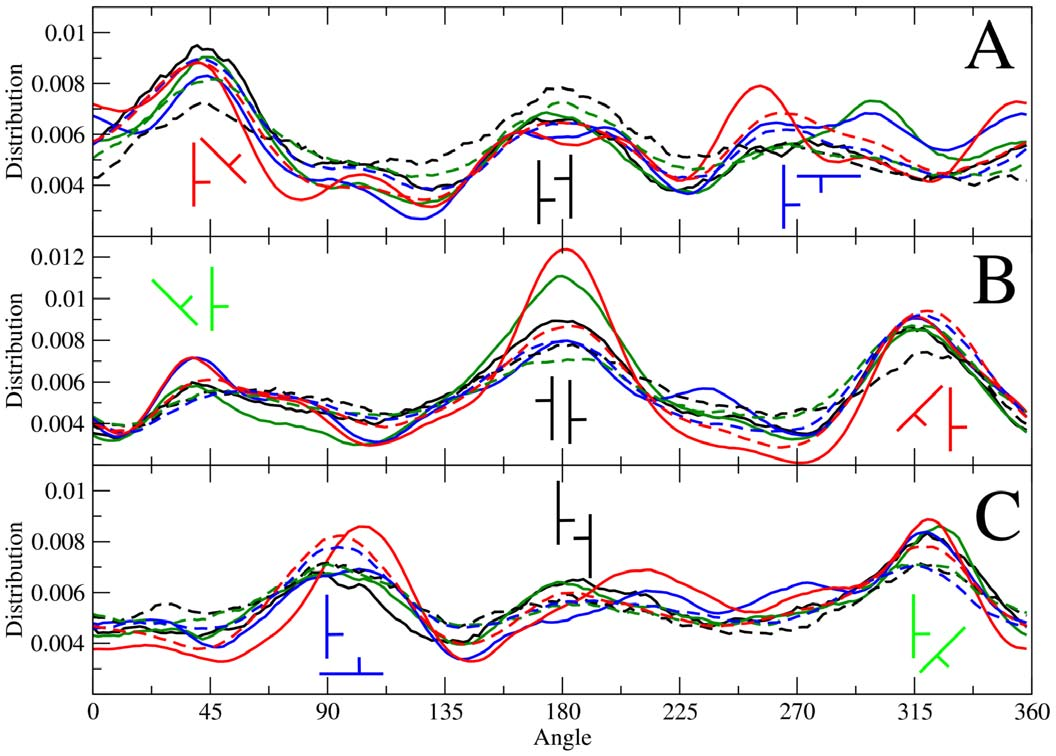
Cholesterol orientation around other cholesterol in the second coordination shell. Angle distribution for cholesterol pairs corresponding to the three preferred locations displayed in the Chol-Chol 2D-density functions. The angle in the *x*-axis is the one formed by the C6–C11 vectors of each cholesterol molecule in the pair. All the curves in a given panel display the same qualitative behavior and here we are interested in characterizing the common trends. Panel (A) refers to the β1 peak. Panel (B) contains the data for the α peak. Panel (C) corresponds to the β2 peak. Solid lines correspond to DSPC systems whereas the dashed curves correspond to DOPC membranes. The color code indicates cholesterol content: 20 mol% (black), 30 mol% (green), 40 mol% (blue), and 50 mol% (red). Simulations with 10 mol% of Chol are not provided due to poor statistics. Preferred configurations are plotted schematically at each distribution maximum. Equivalent configurations are displayed in green, blue, and red, whereas those plotted in black are not equivalent.

We computed the occurrence distribution for the angle formed between the C6–C11 vectors (see Figure 1) of two proximal cholesterol molecules in different sectors of the second Chol-Chol coordination shell. These sectors are defined as follows: NE [22.5°–67.5°], N [67.5°–122.5°], NW [122.5°–157.5°], W [157.5°–202.5°], SW [202.5°–247.5°], S [247.5°–292.5°], SE [292.5°–337.5°], E [337.5°–22.5°], where 0° is in the *x-*direction and angles increase anti-clockwise. This was done by computing separately the relative angle of Chol pairs at a radial distance range of 0.8–1.1 nm in the different angular sectors defined above. The relative angle distributions are plotted in Figure 7 for the three preferred locations in the Chol-Chol 2D-density function (β1(NE[22.5°–67.5°]), α(W[157.5°–202.5°]), and β2(SE[292.5°–337.5°])) for DSPC and DOPC species and different cholesterol concentrations. In all cases, we observed three maxima corresponding to the preferential relative orientations. These nine preferred configurations are schematically depicted in Figure 7, but it is important to notice that some of them are equivalent: the first peak for the α location is equivalent to the third peak in the β2 sector. Next, the third peak in α is equivalent to the first peak in β1 and finally the third peak in β1 is equivalent to the first peak in β2. Essentially, only six different independent configurations are present.

## Discussion

In this article we present new evidence concerning the extraordinary ordering capacity of cholesterol in membranes. Our findings show clearly that the structural characteristics of cholesterol are largely responsible for its ordering abilities. Its small headgroup and the rigid ring structure prevent almost all direct sterol-sterol contacts. This helps cholesterol to avoid the exposure of hydrophobic membrane regions to water which would give rise to a very unfavourable contribution to the free energy. As a result, we often found a PC molecule between two sterol molecules; the big polar head of a PC can effectively shield cholesterol from water. This description, which constitutes the core of the Umbrella model [8], [12], is fully supported by our radial distribution functions presented in Figures 2 and 3. In contrast, the other two existing models accounting for the microscopic cholesterol surrounding, the Condensed Complex model [10] and the Superlattice model [11], are not supported by our data. The Condensed Complex model, which accurately describes cholesterol's behavior in monolayers by the suggested formation of chemical complexes between cholesterol and phospholipids, does not match with our results. Instead of a fixed structure expected for a complex, we observed flexible and transient structures for interactions between cholesterol and the surrounding phospholipids and other cholesterols. As for the Superlattice model, the size of our membranes does not allow a proper comparison and hence we cannot validate the model.

One of the most important observations described in this paper is the particular molecular organization of lipids and cholesterol molecules around a cholesterol molecule. We have shown that off-plane cholesterol methyl groups determine the positional and orientational ordering of PCs around Chol, which, in turn, induce a triangular symmetry for the location of Chols in the second coordination shell with well-defined relative orientations. Splitting the β-face into two sub-faces is fundamental for this ordering mechanism and its effect is remarkable considering the (lateral) fluid nature of the system. This property of cholesterol has not previously been described or discussed in the literature. This finding can be highlighted by the comparison of the properties of Chol and Dchol. The lack of off-plane methyl groups in Dchol structure results in a clear difference in the sterol-sterol 2D-density plots: Chol can clearly be seen as promoting the formation of (dynamic) ‘triangular’ networks whereas Dchol is organized in ‘linear’ arrays (Figure 5). ‘Triangular’ configurations are able to, eventually, expand into 2D sterol-rich regions. Moreover, comparison of the membrane areas between DSPC/Chol and DSPC/Dchol membranes reveals a counterintuitive finding: although Dchol allows a closer lateral approach (due to the absence of C18 and C19), the membrane area at moderate sterol concentrations (for the same sterol content) is larger in DSPC/Dchol than in DSPC/Chol [29]: the equilibrium membrane area for the simulated DSPC/Chol membrane is 39.90 nm^2^ at 20 mol%, whereas the DSPC/Dchol membrane occupies 40.75 nm^2^ at the same sterol fraction. Again, a three-fold symmetry in the lipid arrangement (Chol) is able to condense the membrane more in a 2D-system than a two-fold configuration (Dchol). At high sterol concentrations (i.e. 40 mol%) this situation is inverted due to the increase of sterol-sterol contacts: Dchol dimers are easier to pack laterally than Chol ones due to the absence of off-plane methyl groups.

The Chol biosynthetic pathway shows a systematic removal of methyl groups from the steroid ring. Each removal step optimizes sterol properties in terms of ordering and condensing effects [17]. This observation could lead to the erroneous conclusion that the remaining methyl groups are just evolutionary fossils. However, recent studies have shown that their presence regulates the sterol ring tilt angle in the bilayer [23], [29], and the interaction with double bonds of neighboring lipid acyl chains [21], [22]. Both factors are important in determining a sterol's ability to condense the bilayer area.

There is also reason to discuss experimental observations of membranes with lanosterol. As previously commented, lanosterol is the first sterol in the 19-step biosynthetic pathway to cholesterol [58], whose membrane properties are well characterized experimentally [59]. In lanosterol, the three-fold symmetry is broken by the additional two methyl groups located on the α-face of the steroid ring. The physical properties of lanosterol have important differences compared to those of cholesterol. First, lanosterol's condensing and ordering effects are significantly reduced as compared to cholesterol. Most importantly, however, unlike cholesterol, lanosterol is unable to promote the formation of the liquid ordered phase over a wide range of temperatures and concentrations [59], [60]. Cholesterol is able to do this even at physiological concentrations (>20 mol%).

Another sterol is coprostanol, a product of cholesterol biodegradation, obtained by the reduction of the double bond. Coprostanol differs from other sterols by a *cis* connection between rings A and B. Consequently, the steroid ring in coprostanol is not planar, and both coprostanol and lanosterol therefore lack a flat ring face. In membranes coprostanol has a characteristic phase behavior that differs from other sterols: instead of liquid-liquid coexistence, a liquid-solid coexistence region is observed [60], [61]. Whether both observations are related to the lack of three-fold symmetry remains to be established, but in the light of the results presented here this is the most likely hypothesis. Ergosterol, with the same three-fold symmetry as cholesterol is known to enable the formation of a liquid ordered phase [13], thus supporting this idea.

The above discussed organization of cholesterol surroundings and its consequences have important implications for the development of coarse-grained models for cholesterol [62]. Despite the undeniable utility of coarse-grained approaches to achieve longer length and time scales in MD simulations, some molecular detail is lost, and this may imply certain limitations. The proposed coarse-grained models for cholesterol [62] are essentially symmetric due to the absence of off-plane methyl groups and are therefore unable to describe the three-fold symmetry that our atomistic simulations have revealed. Instead, our results suggest that they would follow a behavior similar to the flat cholesterol (Dchol) considered in the present work. Given this, we suggest that the molecular description of cholesterol in any coarse-grained model should account for the breaking of symmetry between the two cholesterol faces since we have demonstrated that this feature is fundamental for its ordering abilities. It is likely that this feature is the more important the higher is the concentration of cholesterol in a membrane.

The novelty of our results also stems from the identification of a collective/cooperative action of cholesterol molecules in their membrane ordering ability. Analysis of the molecular orientations revealed that cholesterol clearly promotes facing' (θ_90_ = 90°) configurations, especially at higher cholesterol concentrations, resulting in a *sandwiching*-like pattern – two cholesterol molecules *sandwich* a phospholipid. This configuration is found to be more likely for saturated than unsaturated lipids in agreement with cholesterol's higher tendency to order and condense saturated than unsaturated lipids. We propose this cooperative action of two cholesterol molecules as one of the leading molecular mechanisms for the formation of the liquid-ordered phase; the dynamic and cooperative nature is the main reason for why the liquid-ordered phase is not observed at small cholesterol concentrations. It only emerges for Chol concentrations larger than about 10 mol% where the collective self-organization of Chol molecules emerges spontaneously.

We also would like to briefly address the issue of the cholesterol chemical potential. Models and experiments have shown jumps in the cholesterol chemical potential [8]. These jumps are indicators of formation of larger stable structures in membranes, but there is currently no full agreement between the different models and experiments. Considering that the determination of the cholesterol chemical potential and free energy properties from atomistic simulations is a major study of its own, and comparison of the results to all previous findings on equal footing would likely require a great deal of discussion, we leave this topic to a follow-up publication.

Summarizing, the results presented and discussed in this work highlight the importance of cholesterol's unique molecular structure in promoting the formation of transient and local three-fold cholesterol-cholesterol structures that facilitate the formation of the liquid-ordered phase. In this spirit it seems obvious that cholesterol molecules do not function alone but they do so in a co-operative manner, highlighting the importance of understanding collective ordering phenomena in membranes. Furthermore, the present findings have a role to play in functions of raft-associated membrane proteins: as the lipid:protein ratio in native membranes is usually 50∶1 [63], [64], and cholesterol concentration is typically about 30 mol%, the cholesterol:protein ratio in raft-like cell membranes is roughly 20∶1. To understand the role of cholesterol-membrane protein interactions in lipid rafts, one also has to understand the role of self-organization of cholesterol.

## Supporting Information

Text S1 Cholesterol contacts and lipid orientation in the first coordination shell.
(0.04 MB DOC)

Figure S1 In-plane distribution of cholesterols around other cholesterols in DSPC bilayer with 50 mol% Chol. Two-dimensional density distributions for Chol molecules around a tagged Chol for a DSPC bilayer with 50 mol% Chol. The same axis system as in Figure 3B (main text) has been used.
(1.33 MB TIF)

Figure S2 Cholesterol orientation in the first Chol coordination shell. Representation of the five major cholesterol conformations observed in the first cholesterol coordination shell. In black the central cholesterol is shown (oriented as in the two-dimensional density distributions), and around it, the position and orientation of the surrounding cholesterol molecules in the observed configuration are presented. Since some configurations can be easily exchanged along the simulation trajectories, they have been plotted together and considered as the same conformation. The color of the cholesterols indicates an estimation of the probability of each conformation, ordered from the higher to the lower: orange, red, blue, and finally green. The exchange of roles between the central and the surrounding cholesterols leads to identical configurations as they are the same molecule.
(0.05 MB TIF)

Figure S3 Lipid orientation around Chol and Dchol in the first coordination shell. Distribution profiles of the co-localization angles for all simulated systems for lipids inside the first coordination shell of cholesterol. These profiles were computed separately for lipids in each angular sector (NE [22.5°–67.5°], N [67.5°–122.5°], NW [122.5°–157.5°], W [157.5°–202.5°], SW [202.5°–247.5°], S [247.5°–292.5°], SE [292.5°–337.5°], E [337.5°–22.5°], where 0° is placed in the x direction as defined in Figure 1 of the main text). Color code indicates the angular sector. The graphical representation of the co-localization angle is provided next to the graphs, which correspond to the angle between the green and blue arrow as shown. The θ90 representation is used to represent the co-localization angle in all plots (θ90 = 0° corresponds to collinear, θ90 = 45° to diagonal and θ90 = 90° to ‘facing’ orientations).
(1.40 MB TIF)
