# Supplementary material for: Cholesterol Induces Specific Spatial and Orientational Order in Cholesterol/Phospholipid Membranes
Source: PLoS One. 2010 Jun 17;5(6):e11162. doi: 10.1371/journal.pone.0011162 (PMC2887443; doi:10.1371/journal.pone.0011162)
Supplement: Text S1 — Cholesterol contacts and lipid orientation in the first coordination shell. (0.04 MB DOC) [file pone.0011162.s001.doc]

Cholesterol Induces Specific Spatial and Orientational Order in Cholesterol/Phospholipid Membranes

Hector Martinez-Seara, Tomasz Róg, Mikko Karttunen, Ilpo Vattulainen and Ramon Reigada

**Supporting Information**

**Cholesterol contacts in the first coordination shell**

At large cholesterol fraction, the occurrence of direct Chol-Chol contacts is increased as a consequence of cholesterol crowding. The particular configuration of such Chol-Chol structures reveals interesting features. Figure S1 shows the two-dimensional density distribution for DSPC system with 50 mol% of cholesterol. Comparing this figure to the same distribution for the 20 mol% Chol case in Figure 3B (main text), a substantial increment of the occurrence of cholesterol in the first coordination shell is observed. Figure S1 reveals the existence of three preferred approaching directions for direct Chol-Chol contacts at the following angles: [-10º-10º], [80º-100º] and [240º-270º]. These configurations lead to the three peaks observed at short distances in the Chol-Chol radial one-dimensional distributions plotted in Figure 2 (main text). The relevance of these preferred locations dramatically decays at lower cholesterol concentrations. In fact, they are imperceptible for cholesterol fractions lower than 30 mol% for the DSPC system and 40 mol% in the DOPC systems.

Not only positional ordering between cholesterols in direct contact is found but also orientational ordering. We have conducted the same analysis as described in the main text of the article, here for this first coordination shell. It is important to notice that compared to the structures in the second coordination shell, these ones are significantly better resolved; namely, the orientational peaks are higher and narrow than those for the second coordination shell (Figure 7). This can be interpreted as significantly more restricted configurations. The obtained preferential configurations are summarized and detailed in Figure S2. Some configurations resemble the ones observed in the second coordination shell (for instance those labeled as A, C and D), but others are different. It is also interesting to notice that the preferred orientational configurations correspond to the most populated sectors [-10º-10º], [80º-100º], whereas the sector [240º-270º] is more ‘flexible’ and does not contribute with any defined Chol-Chol relative orientation.

At this point one may wonder whether the specific Chol-Chol configurations reported so far may correspond to precursors or initial nucleation structures for subsequent crystallization phenomena. This issue is addressed by comparing the configuration obtained here with those displayed in the three-dimensional cholesterol crystals. This comparison has been performed with the structures provided by Shieh et al. [1] and Hsu et al. [2] We have found little correlation between their configurations and ours. Actually, only the configuration D in Figure S2 is found in the provided experimental crystal structures. Additionally, Craven et al. [3] suggest that in the crystal structure of cholesterol monohydrate some configurations are likely to present the out-of-plane methyl groups within the van der Waals distance. This could suggest the existence of configurations similar to the A and D conformations in Figure S2. Further, there is no possibility for a direct comparison of our two-dimensional interacting cholesterol structures with those of three-dimensional crystals. As shown in previous publications [4], the height of cholesterol in the membrane is almost constant so cholesterols in direct contact interact approximately at the same level. This is not true in crystal structures where cholesterols are placed at different levels of their principal molecular axis in order to optimize packing. Furthermore, the two systems represent completely different environments as membranes contain PC molecules. Therefore, conclusive agreement can not be achieved with three-dimensional cholesterol crystals. Moreover, experimental knowledge of the molecular arrangement of two-dimensional cholesterol crystals in lipid membranes [5] is not available for comparison with our results.

Concerning the cholesterol crystallization issue we have also computed the life-time of the Chol-Chol structures reported in Figure S1. To do so, temporal autocorrelation functions of direct Chol-Chol contact occurrence (defined within the distance of the first coordination shell) are computed, and the time needed to lose the correlation is calculated. For the case in Figure S1, the mean life-time of Chol-Chol structures is found to be of the order of a few nanoseconds, implying that they can not be conjectured as precursors of cholesterol crystals. It seems that any correspondence of our Chol-Chol structures to crystallization phenomena is merely speculative. Actually, the maximum cholesterol fraction in our simulations is 50 mol% while the experimental solubility limit of cholesterol in bilayers of saturated lipids with long chains is well above 50 mol% [6,7]. Actually, for phosphatidylcholine lipid bilayers, the cholesterol solubility is about 67 mol%. Cholesterol crystallization phenomena has been reported in some experiments above 25 mol% Chol, but McElhany et al. [8] have pointed out that these phenomena are probably due to kinetic (not thermodynamic) effects that arise during the preparation process of the binary mixture. Therefore, we conclude that we have not observed crystallization of cholesterol in our simulations, as expected.

**Orientation of lipids in the first Chol coordination shell**

Figure S3 shows that the existence of preferential lipid orientations around cholesterol is not highly dependent on the angular sector where the lipid is located at low sterol fractions (< 30 mol%) in DSPC membranes and in most fractions in DOPC bilayers (only at 50 mol% some differences are observed). However, at fractions larger than 20 mol% Chol in the DSPC systems, the NE and SE sectors display more marked selective profiles.

**REFERENCES**

^1^ Shieh H-S, Hoard LG, Nordman CE (1981) The structure of cholesterol. Acta Cryst B 37: 1538-1543.

^2^ Hsu L-Y, Kampf JW, Nordman CE (2002) Structure and pseudosymmetry of cholesterol at 310 K. Acta Cryst B 58: 260-264.

^3^ Craven BM (1976) Crystal structure of cholesterol monohydrate. Nature 260: 727-729.

^4^ Martinez-Seara H, Róg T, Pasenkiewicz-Gierula M, Vattulainen I, Karttunen, et al. (2008) Interplay of Unsaturated Phospholipids and Cholesterol in Membranes: Effect of the Double-Bond Position. Biophys J 95: 3295-3305.

^5^ Mason RP, Tulenko TN, Jacob RF (2003) Direct evidence for cholesterol crystalline domains in biological membranes: role in human pathobiology. Biochim Biophys Acta 1610: 198-207.

^6^ Huang J, Buboltz JT, Feigenson GW (1999) A Microscopic Interaction Model of Maximum Solubility of Cholesterol in Lipid Bilayers. Biochim Biophys Acta 1417: 89-100.

^7^ Ali MR, Cheng KH, Huang J (2007) Assess the nature of cholesterol-lipid interactions through the chemical potential of cholesterol in phosphatidylcholine bilayers. Proc Natl Acad Sci USA 104: 5372-5377.

^8^ McMullen TPW, Lewis RNAH, McElhaney RN (2009) Calorimetric and spectroscopic studies of the effects of cholesterol on the thermotropic phase behavior and organization of a homologous series of linear saturated phosphatidylglycerol bilayer membranes. Biochim Biophys Acta 1788: 345-357.
