# Supplementary figures and images for: Cholesterol Induces Specific Spatial and Orientational Order in Cholesterol/Phospholipid Membranes
Source: PLoS One. 2010 Jun 17;5(6):e11162. doi: 10.1371/journal.pone.0011162 (PMC2887443; doi:10.1371/journal.pone.0011162)

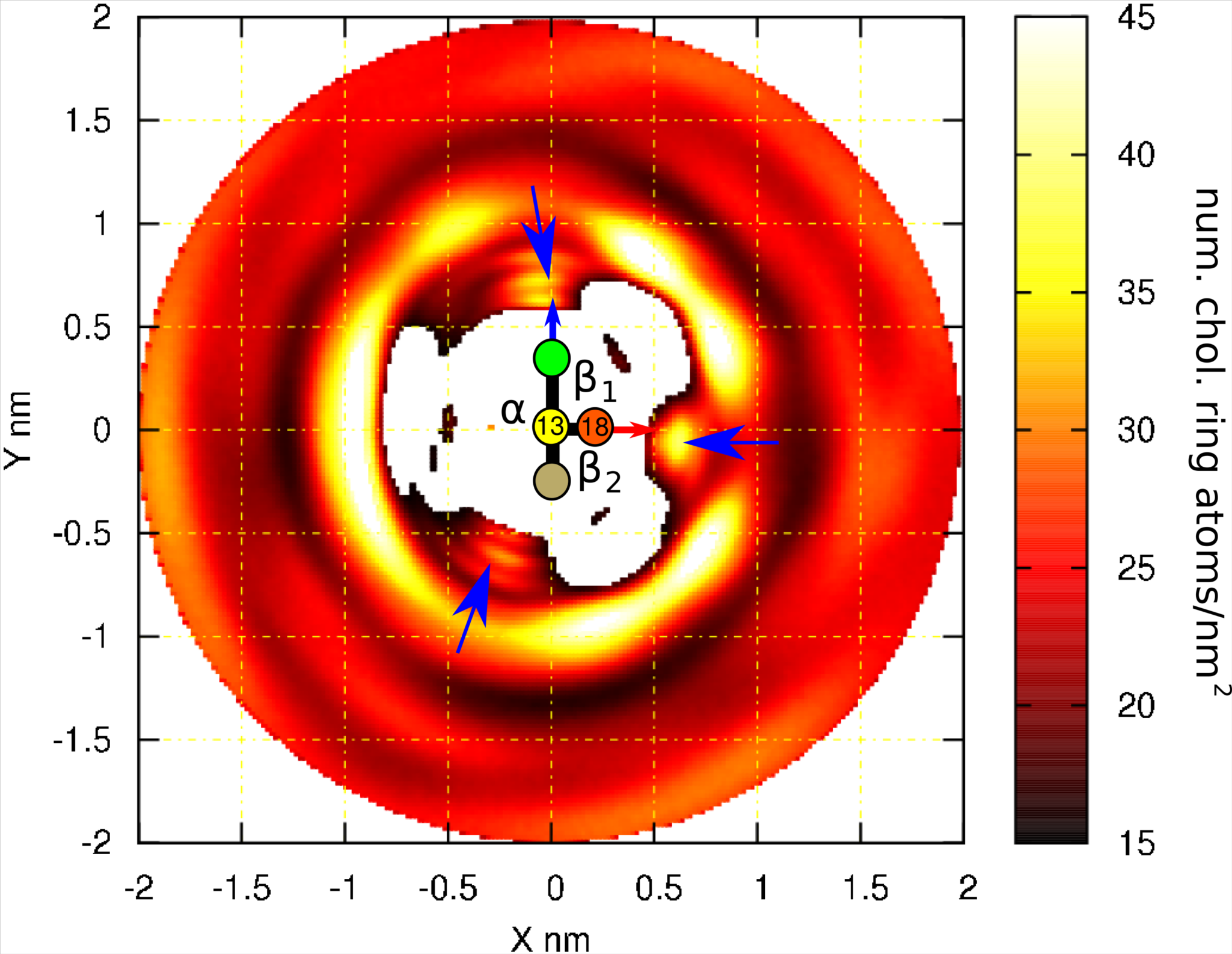

Supplement: Figure S1 — In-plane distribution of cholesterols around other cholesterols in DSPC bilayer with 50 mol% Chol. Two-dimensional density distributions for Chol molecules around a tagged Chol for a DSPC bilayer with 50 mol% Chol. The same axis system as in Figure 3B (main text) has been used. (1.33 MB TIF) [file pone.0011162.s002.tif]

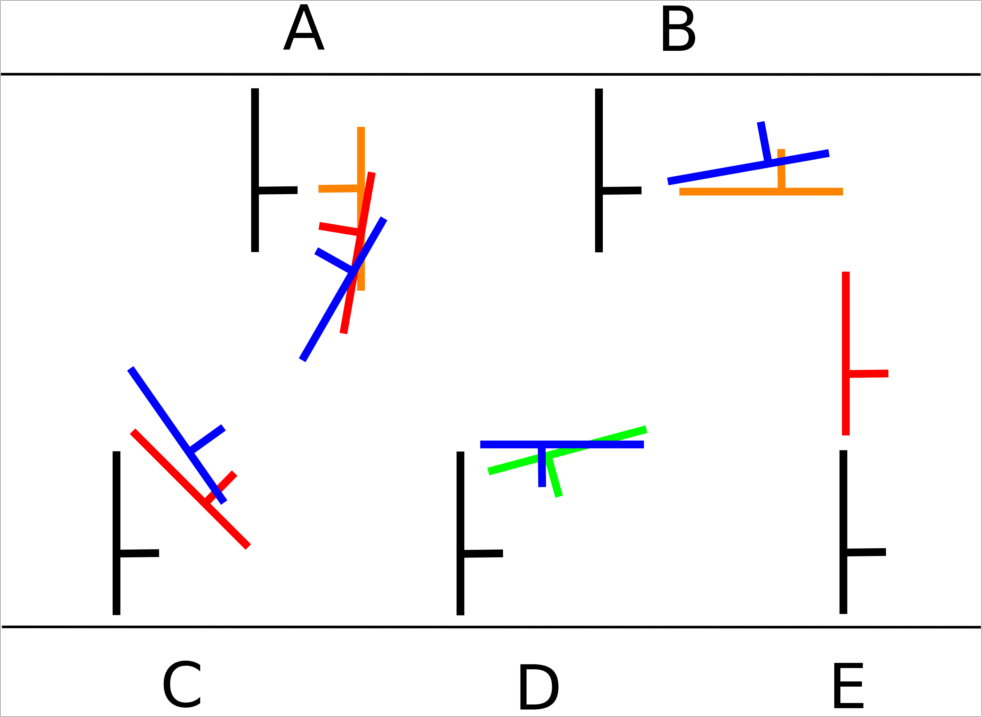

Supplement: Figure S2 — Cholesterol orientation in the first Chol coordination shell. Representation of the five major cholesterol conformations observed in the first cholesterol coordination shell. In black the central cholesterol is shown (oriented as in the two-dimensional density distributions), and around it, the position and orientation of the surrounding cholesterol molecules in the observed configuration are presented. Since some configurations can be easily exchanged along the simulation trajectories, they have been plotted together and considered as the same conformation. The color of the cholesterols indicates an estimation of the probability of each conformation, ordered from the higher to the lower: orange, red, blue, and finally green. The exchange of roles between the central and the surrounding cholesterols leads to identical configurations as they are the same molecule. (0.05 MB TIF) [file pone.0011162.s003.tif]

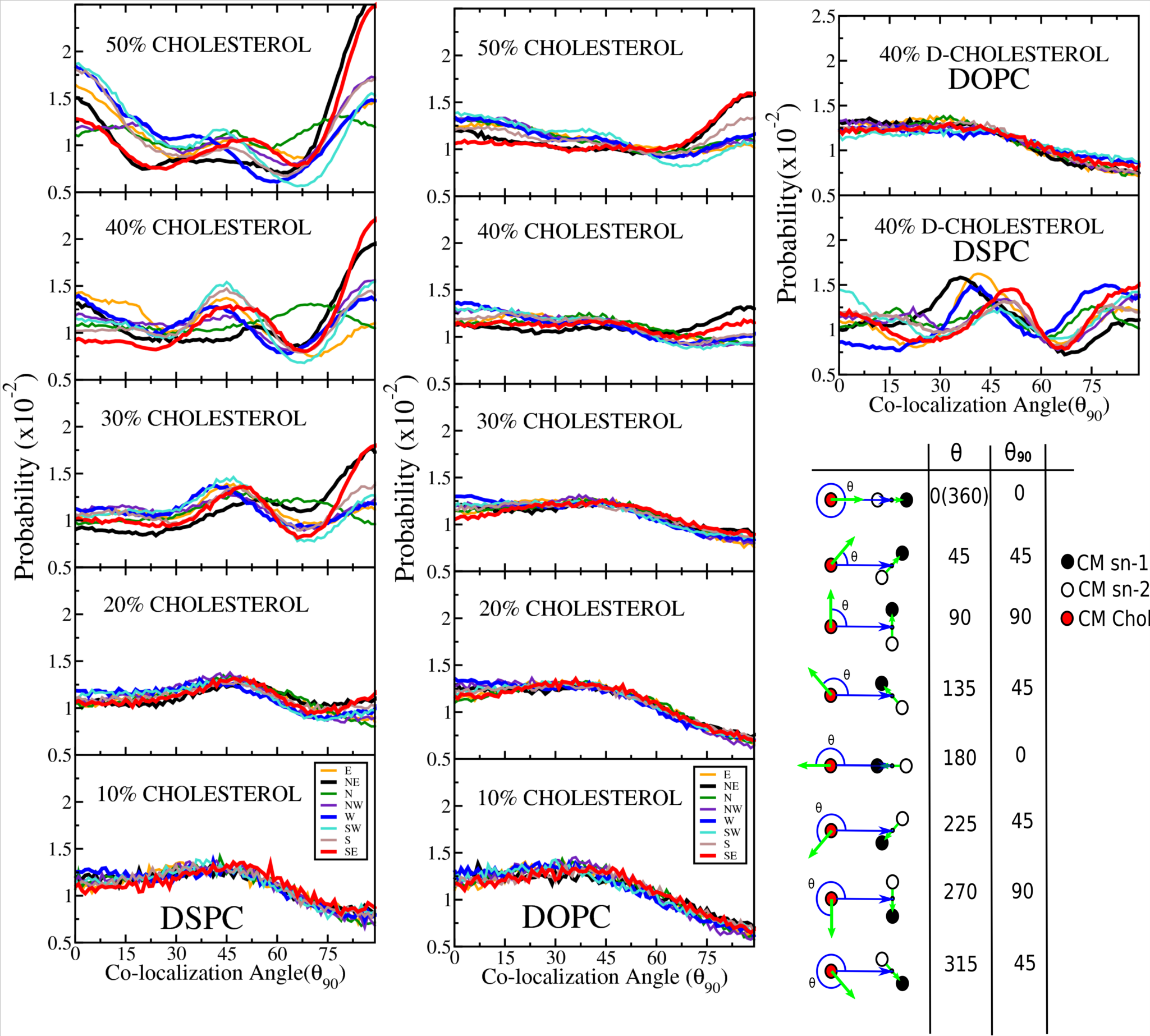

Supplement: Figure S3 — Lipid orientation around Chol and Dchol in the first coordination shell. Distribution profiles of the co-localization angles for all simulated systems for lipids inside the first coordination shell of cholesterol. These profiles were computed separately for lipids in each angular sector (NE [22.5°–67.5°], N [67.5°–122.5°], NW [122.5°–157.5°], W [157.5°–202.5°], SW [202.5°–247.5°], S [247.5°–292.5°], SE [292.5°–337.5°], E [337.5°–22.5°], where 0° is placed in the x direction as defined in Figure 1 of the main text). Color code indicates the angular sector. The graphical representation of the co-localization angle is provided next to the graphs, which correspond to the angle between the green and blue arrow as shown. The θ90 representation is used to represent the co-localization angle in all plots (θ90 = 0° corresponds to collinear, θ90 = 45° to diagonal and θ90 = 90° to ‘facing’ orientations). (1.40 MB TIF) [file pone.0011162.s004.tif]
